# Supplementary material for: Correlation between 146S Antigen Content in Foot-and-Mouth Disease Inactivated Vaccines and Immunogenicity Level and Vaccine Potency Alternative Test Methods
Source: Vet Sci. 2024 Apr 8;11(4):168. doi: 10.3390/vetsci11040168 (PMC11053669; doi:10.3390/vetsci11040168)

Supplementary Table S1 Raw data of 146S/dose and PD_{50}

146S（μg/dose）	PD_{50}	146S（μg/dose ）	PD_{50}	146S（μg/dose ）	PD_{50}
4.724	7.05	5.884	7.49	10.136	10.81
6.066	7.05	5.578	7.49	15.612	10.81
5.048	7.05	6.746	9	15.614	11.84
6.036	7.19	7.416	9	14.904	11.84
5.646	7.19	6.746	9	15.426	13.59
5.198	7.19	6.708	10.32	10.802	13.59
4.068	7.49	7.546	10.32	11.812	13.59
5.574	7.49	10.022	10.32	12.718	13.59
16.4	13.59	16.546	15.59	18.542	15.59
15.392	13.59	17.688	15.59	19.052	15.59
13.14	13.59	17.732	15.59	21.6	15.59
14.944	13.59	18.554	15.59	22.186	15.59
15.806	13.39	19.074	15.59	38.898	15.59
16.736	13.59	20.188	15.59	29.058	15.59
15.806	13.59	18.704	15.59	15.632	13.59
18.426	15.59	/	/	/	/

Supplementary Figure S1. Qualification rates of antibody titers following primary immunization at different times with varying 146S vaccine concentrations

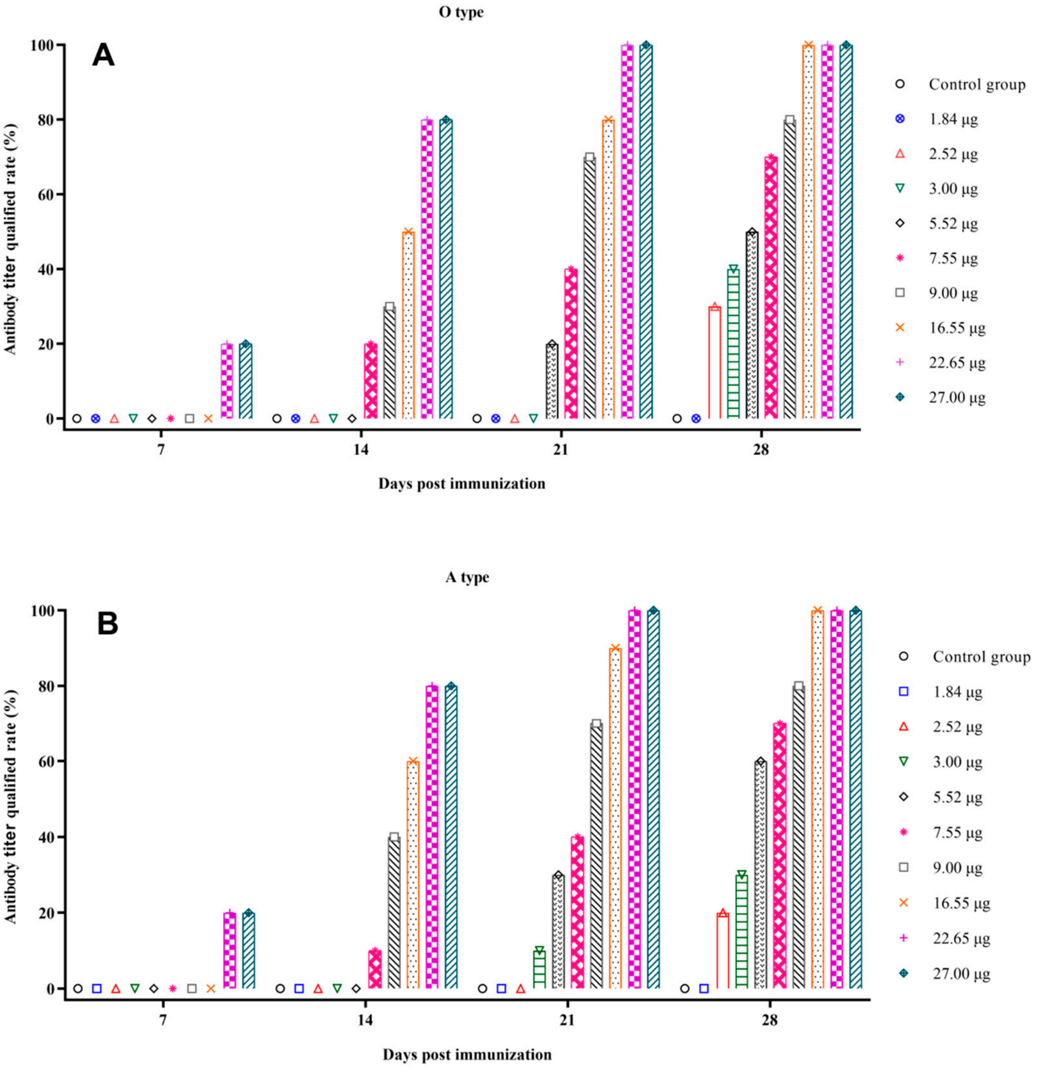

Supplement: Supplementary file 1 [file vetsci-11-00168-s001.zip › vetsci-2925016-supplementary.pdf]
